# Mental health and psychosocial support services in primary health care in Nepal: perceived facilitating factors, barriers and strategies for improvement

**DOI:** 10.1186/s12888-020-2476-x

**Published:** 2020-02-13

**Authors:** Nawaraj Upadhaya, Upasana Regmi, Dristy Gurung, Nagendra P. Luitel, Inge Petersen, Mark J. D. Jordans, Ivan H. Komproe

**Affiliations:** 1 Transcultural Psychosocial Organization Nepal, Kathmandu, Nepal; 2 Centre for Rural Health, College of Health Sciences, University of KwaZulu-Natal, Durban, South Africa; 3 Department of Research and Development, War Child, Amsterdam, the Netherlands; 4 Centre for Global Mental Health, Institute of Psychiatry, Psychology and Neuroscience, King’s College London, London, UK; 5 Department of Research and Development, HealthNet TPO, Amsterdam, the Netherlands; 6 Utrecht University, Utrecht, the Netherlands

**Keywords:** Mental health and psychosocial support, Primary health care workers, Nepal, Facilitating factors, Barriers

## Abstract

**Background:**

The barriers and facilitating factors for integrating mental health into primary health care have been well documented in the literature, but little is known about the perspectives of primary health care workers (who provide integrated mental health care) on barriers and facilitating factors of the health system for scaling up mental health interventions in low and middle income countries. This study aimed to explore these perspectives of primary health care workers within the health system, and identify possible strategies to optimize the integration of mental health in primary health care.

**Methods:**

The study was conducted in the Chitwan district of Nepal with 55 purposively selected primary health care workers representing prescribers (*N* = 35), non-prescribers (*N* = 12) and Female Community Health Volunteers (*N* = 8). Using a semi-structured interview guide, experienced qualitative researchers collected data between September 2016 and May 2017. The interviews were audio-taped, transcribed and then translated into English. The transcripts were coded using Nvivo 10 software and themes were generated for the thematic analysis.

**Results:**

According to the health workers, the facilitating factors for scaling up mental health services in primary health care setting in Nepal included; (1) availability of guidelines, protocols and awareness raising materials, (2) provision of supervision, (3) referral systems being in place, (4) patient record keeping, (5) community sensitizations and home visits, and (6) provision of psychosocial counseling. The barriers identified included; (1) shortage of psychotropic medicines, (2) lack of private space for counseling, (3) workload and health workers’ grievances regarding incentives, and (4) perceived stigma causing dropouts.

**Conclusions:**

The findings suggest that implementation of mental health services through primary health care workers in resource-poor setting is possible when health system level barriers are addressed and facilitating factors are strengthened. In order to address these barriers the health workers suggested a few strategies which included; ensuring dedicated staff available at health facility, allocating dedicated and confidential space for counseling, improving on incentives and motivational benefits to existing health staff, organizing policy level advocacy for mental health, improving medicine supply chain management and strengthening systems for supervision, referral and mental health information management.

## Background

Mental, neurological and substance use (MNS) disorder account for 10.4% of global disability adjusted life years (DALYs) [1]. The global burden of disease study 2015 reported that the depression and anxiety were the third and ninth leading causes of disability respectively [2]. Yet, a small percentage of people who need mental health care have access to mental health treatment, the so-called treatment gap. In low and middle income countries this treatment gap has reached to nearly 90% [3]. A study including 21 countries found that only 1 out of 27 people with major depressive disorder received minimally adequate treatment [4].To address the unmet need of people with mental health problems, there has been increasing calls to scale-up mental health services which means increasing the coverage of services, the range of evidence-based services and strengthening health systems to facilitate service delivery [5]. In 2008, WHO launched the mental health gap action program (mhGAP) in primary health care to scale-up cost-effective interventions for MNS disorders through the training and supervision of primary health care workers based on a task-sharing approach [6]. This task-sharing approach (mobilizing primary health workers in the diagnosis and treatment of common mental disorders) is perceived to be feasible to implement when other components of service delivery such as supply of drugs, continued clinical supervision by specialists, and clear administrative and governance procedures are put in place [7].

However, several studies also have documented barriers for scaling up mental health services in low- and middle-income countries. Some of those barriers include lack of priority and financial resources for mental health care, absence of decentralization mechanisms for mental health, and the low number of primary health workers trained and supervised in mental health [8]. Other barriers include failure of the primary health care systems to detect people with mental illness, problems in motivating primary health care staff to provide mental health services, the high concentration of mental health services and human resources in tertiary hospitals, lack of mental health and psychosocial interventions in the community and problems providing mental health services at the primary health care settings [9]. Nevertheless, there are also some promising developments for integration of mental health in primary health care in low resourced settings. For example, regular training and mentorship of primary health care nurses in Rwanda and Ethiopia has helped in integrating mental health services at the community level [10, 11]. Similarly, in Nepal, the national mental health policy, Nepal Health Sector Plan and multi-stakeholder action plan for non-communicable diseases, all promote the integration of mental health in primary health care [12].

In the context of integrated mental health care, contrary to disease-focused view, the person focused and population based perspective as suggested by Valentijn and colleagues [13] can link health and social systems, both of which affect/address mental health wellbeing of person and the populations. The decision to access and use health services is determined by the presence of individual and community level enabling resources [14]. In case of mental health, one of the enabling resources is the organization of mental health care, the way mental health care is organized influences its access and use. Secondly, the health system factors such as health infrastructure, institutional procedures and regulations as well as human and financial resources affect the access and utilization of health services [15].

For our study, the presence of enabling resources at the health facility are the facilitating factors for the integration of mental health into primary health care. Likewise, system level barriers are the lack of enabling resources or lack of their proper management at the health facility. These barriers are responsible for low percentage of realized access (service utilization).

The barriers and facilitating factors for integrating mental health into primary care have been well documented, but mainly at the national level [16, 17]. Little is known about the perspectives of primary healthcare workers (who provide integrated mental health care) on health system level barriers and facilitating factors for scaling up mental health interventions. This information is of critical importance to inform if and how mental health service delivery mechanisms can be scaled up, as these healthcare workers will take on a new burden of care in mental health service provision if a task-shifting approach is used. In rural areas in countries such as Nepal, mental health is highly stigmatized and neglected not only by the government but also by other non-governmental and community structures [18]. To address this information gap, the present study was guided by the following study aims and research questions.

### Study aims

- To conduct an assessment of health system synergies/implications of integration of mental health into primary health care in low resources setting.
- To identify system level processes, facilitators and barriers for the integration of mental health in primary health care setting.
- To understand interventions/mechanisms put in place to address system level bottlenecks.
- To explore strategies to address barriers and strengthen facilitating factors.
- To understand whether and how integrated mental health services may have increased the burden of care at the primary health care level.

### Research questions

- What are the existing facilitating factors for the integration of mental health into primary health care?
- What are the system level factors or bottlenecks that impede integration of mental health into primary health care?
- What interventions/mechanisms were put in place to address these bottlenecks?
- What are perspectives on whether integrated mental health care has led to a strengthening of the overall provision of chronic care?
- What are perspectives on whether and how integrated mental health services may have increased the burden of care at the primary health care level?

## Methods

### Study setting and context

Chitwan district is located in Southwestern Nepal. As per 2011 census Chitwan had a total population of 579,984 with 48% male and 52% female. The 27% of the population in Chitwan live in urban areas [19]. Chitwan district is known for its medical facilities, the district headquarter Bharatpur has both government and private hospitals where people from mostly western part of Nepal come for services. Outside of the district capital, however, only government primary health care workers are providing general health services mostly the antenatal and post-natal care, immunization and treatment of common waterborne and seasonal diseases. A community survey conducted in the same district found that 11.2% of the sample screened positive for depression and 5.0% screened positive for alcohol use disorder [20]. Similarly, the health facility based study suggested that nearly 19.6% of females and 11.3% of males had depression [21]. This study was conducted in Chitwan where a district mental health care package was implemented since 2011 in 12 health facilities and scaled-up to all health facilities of Chitwan district in 2016 through a government-NGO pilot mental health project called PRIME (program for improving mental health care) [22], complemented by another project called Emerald (Emerging mental health systems in low and middle income countries) [23]. Through PRIME, three types of primary health workers (prescribers, non-prescribers and Female Community Health Volunteers) were trained on several aspects of primary mental health care. The details of the project components and training programs are published elsewhere [24]. In brief, all health facility staff received 4 days training on basic psychosocial support and stigma reduction while prescribers (medical officers, health assistants and community medical assistants) received additional 5 days training on pharmacological treatment (diagnosis, drug prescription and side effect management) and non-prescribers (nurses and midwives) received additional 5 days of training on specific psychosocial intervention protocols for depression and alcohol abuse. The female community health volunteers (FCHVs) received 2 days of training on home-based care, mental health community awareness programs, and a community informant detection tool (CIDT) developed for proactive mental health case detection from the community and referral to the nearby primary healthcare center [25–27]. Table 1 provides the types of health workers, training duration and training topics.

**Table 1:** Training topics and duration

Types of health workers	Training duration	Training topics
FCHVs (female community health volunteers)	2 days	• Anti-stigma program, mass sensitization and awareness raising on mental health and psychosocial problems, referral pathways and available services. • How to provide home based care for people with MNS disorders. • How to identify and refer people with MNS disorder with the help of Community Informant Detection Tool (CIDT).
Non-Prescribers(nurses and midwives)	9 days	• Concepts of basic psychosocial problems and supporting skills. • Anti-stigma program, mass sensitization and awareness raising. • Psychosocial counseling-concepts and skills. • Relaxation exercises and peer support interventions • Brief protocolized psychosocial interventions such as, Healthy Activity Program (HAP) for depression patients and Counseling of Alcohol Program (CAP) for patients with alcohol problems. • Psycho-education on self-care management strategies, stress and anger management techniques.
Prescribers (medical officers, health assistants and community health assistants)	9 days	• Concepts of basic psychosocial problems and supporting skills. • Anti-stigma program, health facility level stigma for mental illness. • Psycho-education on self-care management strategies, stress and anger management techniques. • Assessment, diagnosis and pharmacological treatment of MNS disorders as per mhGAP guidelines. • Common side effects of psychotropic drugs and consequences of inappropriate use of drugs. • Use of treatment plan flow chart and checklist for screening suicidal ideation, depression, epilepsy, psychosis and alcohol use disorders. • Monthly data compilation using data from patient registers. • Drug quantification, storage, recording and drug demand and supply tracking system.

The Emerald project was implemented alongside PRIME and aimed to improve mental health outcomes by strengthening health system performances specially through capacity building (of primary health care workers, policy makers, researchers and servicer users/care givers in several aspects of mental health system strengthening in primary health care setting), exploring sustainable financing for mental health, building governance and leadership structures at national and sub-national levels and establishing functional mental health information system within the government’s health management information systems.

### Sample

All 607 primary health care workers (163 prescribers, 148 non-prescribers and 296 FCHVs) who received training from the PRIME project were eligible to participate in the study because they could provide their experiences and perceptions on opportunities and challenges of scaling up mhGAP based district mental health care package in other districts of Nepal. Of these (607 primary health care workers), the study sample (*N* = 55) was selected purposively, stratified by the above three types of health workers. The prescribers (*N* = 35) included 28 males and 7 females. The respondents for non-prescriber category (*N* = 12) and FCHVs (*N* = 8) were all female as in Nepal these jobs are only for women.

### Data collection instruments and process

For each type of health worker, a separate semi-structured interview topic guide was used to collect the information. Based on the literature review and previous study results, the interview guide was first developed in English by the Emerald consortium. The interview guide was translated into Nepali by experienced researcher. Then a group of researchers looked both English and Nepali versions of the interview guide and discussed whether the translation captures the real meaning of the questions. The group made some changes to address the issues related to clarity, relevance and usefulness of the questions. The draft interview guide was piloted with a few health workers in the project location to assess whether the questions are clearly understood or not. After the pilot the language and flow of questions was changed and some probing questions were added. The final interview guide consisted questions related to diagnosis and treatment of MNS disorders, availability of psychotropic drugs, experiences with the integration of new mental health indicators, administrative and logistical challenges of integrating mental health into primary health care, facilitating factors and barriers for scaling up mental health integration in primary health care setting and unintended consequences of adding mental health responsibility to primary health care workers on top of what they are already doing for patients with physical health problems.

The data collection took place between September 2016 and May 2017. All interviews were conducted in Nepali and audio-taped by a team of researchers with university level education and minimum 2 years of experience in qualitative research. The researchers received training for using the interview topic guides. Each question of the topic guides was discussed among the group of researchers and meaning of each question was explained. Researchers were encouraged to conduct regular self-reflection on their role and critically examine whether knowingly or unknowingly they have influenced the research process during sample recruitment and site selection. They were also encouraged to ask relevant probing questions related to the topic guide, but not to deviate from its overall theme.

### Data analysis

The audio-recorded interviews were first transcribed in the original language (Nepali) by the researchers who took the interview. The transcriptions were then translated into English by professional translators and a few sample translations were crosschecked with the original by the supervisor (NU). To identify themes and associated codes within each theme, two researchers from the research team first read and coded 10% of the interviews separately and generated a coding framework for thematic analysis. The two sets of themes and codes generated by two researchers were shared among a group of researchers of the EMERALD project who were familiar with the design of the study and involved in data collection. Based on discussion, the coding framework was finalized and applied to the transcripts uploaded in qualitative data analysis software, NVivo-10. During the coding process in NVivo-10, the themes and codes were further refined and data were summarized and charted. The summary was exported from NVivo to Excel Spreadsheet and cross-checked for any inconsistencies. When, inconsistencies were found, they were corrected after looking at original transcripts.

To reduce the potential bias of selecting certain types of quotes, two authors (NU and UR) were involved in data analyses which lead to selecting the quotes.

We adopted a thematic analysis framework as described by Nowell and colleagues [28] because each stage of thematic analysis establishes trustworthiness. In thematic analysis there are mainly five stages namely familiarization with data set, identifying initial codes, searching for themes, reviewing themes and defining and naming the themes. These stages helped establish trustworthiness of data analysis and interpretation by giving an opportunity to have prolong engagement with data set, sufficient time to reflect on codes/themes and triangulation with data collection modes. These stages also provided opportunities for peer debriefing, researcher triangulation, reflexive journaling, use of coding frameworks, use of diagraming to make connection to several themes. The stages of thematic analysis helped us in determining the hierarchies of concepts and themes, vetting of themes and sub-themes by team members and team consensus on final themes. The Table 2 provides main result summaries grouped into three themes namely facilitating factors, barriers and strategies for improvement.

**Table 2:** Facilitators, barriers and strategies for improvement

Facilitating factors	Barriers	Strategies for improvement
• Availability of the guidelines, protocols and awareness raising materials • Provision of refresher trainings, clinical supervision, coaching system • Provision of referral system • Provision of patient record keeping system • Provision of community awareness and linkages • Provision of home based care by FCHVs • Efforts in maintaining privacy and confidentiality. • Provision of psychosocial counseling and other protocolized psychosocial interventions • System level co-ordination • Provision of free treatment	• Frequent transfer of trained staff • Lack of separate space for counseling • Limited number of health staffs/ workload • Shortage of medicines time and again (psychotropic drugs) • Health workers’ grievances on incentives/transportation costs • Defaulters in referral as well as in treatment follow up • Patients not going to the referred places • Stigma for people with mental illness • Lack of data captured in national HIMS • Limited awareness about mental health in the community	• Develop provisions for dedicated staff available at health facility at all times • Allocate confidential space for counseling • Improve on incentives/motivational benefits to existing health staff to compensate work burden • Organize policy level advocacy for mental health. • Improve overall drug supply chain management • Improve overall training mechanisms and supervision system • Improve the mental health data collection forms (simplifying the language used in the form) • Strengthen the two-way referral system • Increase the engagement of recovered patients and their family member in stigma prevention programs • Focus upon the factors on scale up and sustainability of the program

## Results

### Facilitating factors

#### Availability of the guidelines, protocols and awareness raising materials

The availability of guidelines and awareness raising materials was thought to be a facilitating factor for mental health service delivery as primary health care workers could refer to those documents when confused about diagnosis and treatment procedures. For example, guidelines are available on suicide screening, adverse effect management, treatment for priority mental disorders. A non-prescriber said, *“When we are busy we might forget some of the points [provided during the training] in that case we use those guidelines..... The guidelines help to identify all the signs and symptoms of the person”.*

Although all primary healthcare workers acknowledged the existence of these materials, there were diverse views on their availability, particularly the information, education and communication (IEC) materials for mental health promotion and awareness raising. Some respondents were of the opinion that they were sufficiently available whereas others thought that availability was limited and that this negatively affected efforts to raise community awareness of mental health.

#### Provision of supervision

The provision of regular clinical supervision was perceived to be helpful by all health workers in course-correction and their continued learning. A non-prescriber said, “*We get knowledge on how we should handle cases and if we have missed anything or made any mistakes then we get the chance to learn about it. This [supervision] is very effective”.*

During the supervision meetings, the practice of seeing the client in front of primary health care workers was thought to be very effective as this provides opportunity for hands on learning. A male prescriber explained this by saying, “…*the doctor tells us how to diagnose the case, whether we should increase or decrease the dose of medicines of particular cases or not”.*

Over time, the supervision system was decentralized from the district to clinical sub-centers, which respondents considered to be beneficial because on site supervision helped to assess the progress in real time and increased the availability of specialist care in primary health care centers, “*This has been helpful because each patient cannot go to Bharatpur* [district headquarter]*, if we conduct meeting in different places, it is easier for them to attend [consultation with psychiatrist]”.*

According to the prescribers, the availability of supervisors [psychiatrists] on phone when primary healthcare workers need to consult them further strengthened the supervision system. One of the female prescribers said, *“In cases where we are confused we can call him [psychiatrist based in Chitwan]. He is available through phone too and he gives us suggestions”.*

#### Referral system being in place

The formal referral pathway implemented in the district was thought to be a facilitating factor as it provided clear guidelines on how and where referrals can be made. For example, the referral pathway began with the Female Community Health Volunteers (FCHVs) who referred the suspected mental health cases to the primary health care center where the prescribers provided medication and non-prescribers provided psychosocial support. Depending upon the case severity and specific needs of the patients, referral was made to the psychiatric doctors based at district hospital or the community counselors.

There was a widely accepted view among the respondents that the referral system was working well. A prescriber shared his experiences saying,“ *It’s very effective. There was one person from Gorkha, her brother was principal of one school in Gorkha but he started drinking from early morning to night. She then brought her brother here and we looked after that case. I thought that the case was little severe and referred it to Doctor [in Bharapur]. Doctor looked at the case and prescribed some medicines. We provided those medicines from health post later. Now that person’s family, everyone is happy with the improvement seen on him. His sister comes to us and says that because of our help things are getting better for them”.*

However, some health workers also said that patients and family members in the beginning complained that the doctors did not give time to referred patients, but that facilitated discussion with doctors and primary health care workers served as a useful facilitating factor to ensure that sufficient care was provided. A male prescriber told: “*at first it was difficult for us to convince them* [patients, to seek care]*. They used to say: ‘why should we go there? We do not have time, money and doctors do not give us time’. Then in monthly meeting we said to the doctors: ‘we only send those cases which we found difficult and you have to give them time otherwise it is very hard for us to work at the community level’ and then they provided good services to the patients”.*

An informal system of back referral took place where the consultant psychiatrist would prescribe those medicines that are freely available at the primary health care facilities and would refer the patients to the health facilities for medicines. Health workers thought this a facilitating factor as patient would not need to spend money to buy the drugs from pharmacy and patients would have more trust on the services provided by primary health care workers. A male prescriber said, *“there are some patients who do not come here and directly go to Bharatpur and Bharatpur hospital refers the patient here”.*

#### Provision of home visits, psychosocial counselling and patient record keeping

Applying home visits by Female Community Health Volunteers (FCHVs) was perceived as helpful as it facilitated treatment adherence by the service users and family members. According to the respondents, the family and community members found it easy to express their problems with FCHVs who were also from the same community. A FCHV said, “*They also said that, they see us (volunteers) as their own family members or their neighbors so they feel easier to share their problems with us.”* The home visit itself turned into an intervention as service users and family members were more cautious with their behaviour and daily practices. A FCHV said, *“those people having severe mental health problems didn’t use to obey their family members but as they were informed that we will be visiting them then they have started reducing the consumption of alcohol”.*

The provision of psychosocial counseling within the health facility was thought to be a facilitating factor as it encouraged health workers to reduce the overuse of medication and implement more psychosocial therapies for people with distress related problems. Patients were provided with psychosocial counseling by non- prescribers taking into account the issue of confidentially. This change was attributed to the increased awareness among the health workers on importance of counseling and other psychosocial support. This was explained by the following interview excerpt of a male prescriber: *“Before we didn’t even have a counseling room. There were problems because of stigma and because we didn’t have counseling rooms that time it was difficult to maintain confidentiality. In our health post it’s not a problem now. We have counseling room”.*

The focus on privacy and health workers efforts to maintain confidentiality encouraged service users and family members to openly express their problems, without any fear of somebody listening to their conversation. This helped in rapport building, treatment adherence and recovery.

Likewise, the respondents found patient record keeping very useful to complete collected information about the patient and that it was available in a separate mental health register, making it easy to find and use the information during the course of the treatment. A non-prescriber explained why this was useful by saying, “*It is useful because we cannot remember the name of all patients and once we open the register we came to know complete history of that patient with the help of OPD [Out Patient Department] number. It is very good* “.

#### Community sensitization

The respondents thought that the community sensitization program and home based care helped reduce social stigma and raise awareness about harmful effects of not treating mental illness. According to them, due to this community linkage, the treatment seeking behaviour improved and more people visited the health post. A female prescriber said, *“We have been able to achieve this because of awareness raised by FCHVs. Because of the FCHVs people with such problems are now coming to health post”.*

The mental health orientation to traditional healers was thought to be a facilitating factor as traditional healers after identifying the sign and symptoms of mental disorders, referred the people to health facility for further treatment. A non-prescriber explained this by saying, “this *program has oriented traditional healers like Dhami, Jhakri of the community as well. Before the implementation of TPO program [pilot mental health project] people used to go to Dhami , Jhakri if they do not have proper sleep, lost the appetite for food, headache; even now though some of the people still practice this behavior but traditional healers send them to the health post which is a great achievement of the program so far”.*

### Barriers

#### Unavailability of trained staff and private space for counselling

Unavailability of sufficient trained health workers in health facility due to their frequent transfers was perceived to be one of the major barriers in mental health service delivery. Legally, the non-prescribers were not authorized to prescribe medication to the patients. Therefore, when there were very few or no trained health workers (prescribers) in health facilities, patients had to be returned or referred to other mental health service providers. A non-prescriber explained this by saying, *“There are two prescribers now here in our health post. They have received training [mental health] provided by the organization so if they are transferred to some other areas then later, there won’t be human resource who have received such knowledge or who have such experience”.*

In some health facilities, especially those with higher client flow, the lack of private space for counseling was one of the barriers as it was difficult to maintain privacy and confidentiality of patients and family members attending the psychosocial counseling sessions. A female prescriber said, “If *the patient flow is high in the health post and in that condition we have to provide counseling in the OPD [out-patient department] room which is very small and difficult to maintain the privacy of the patient”.*

#### Shortage of psychotropic medicines

Shortage of medicine came up as one of the biggest barriers as it created mistrust between the health workers and patients/family members. This was explained by a prescriber who said, *“We taught them [patients] to take medicine and provided it free of cost previously but now if they have to buy those medicines they argue with us, so it is being difficult for us to work at the local level”.* The shortage, however, varied across health facilities. The health facilities with higher client flow were the most affected by the stock out of the medicines. Those who spoke about the unavailability of the drugs said, *“It’s really inconvenient for us. There aren’t adequate medicines. The patients take around one box of medicine, each time they come here. DPHO [District Public Health Office] gives us two boxes of medicine but the patients in need of medicine are around 60”.*

#### Workload and lack of incentives/transportation costs

Workload was thought to be a barrier by some health workers as it gave them extra work and reduced their time for rest. However, the work burden after the introduction of mental health program emerged as a contested issue. Some health workers thought that there was work burden while others said that there was nothing like work burden. Those speaking in favor of work burden argued that mental health patients require more time during consultation so health workers have to work hard to respond to all the patients visiting the health facilities.

A male prescriber alluded, “*When patients with mental health problems come, we have to give them time as most of the time they become restless but while we are giving them time, other people[patients with physical health problems]come and disturb and argue with us. So such problem is there. If we try to give priority to these cases, the other person will die as they might be bleeding, or might be in severe condition. It’s difficult to manage time”.*

The lack of sufficient incentives was perceived by some health workers a barrier as it de-motivated health workers to take on extra work related to mental health service delivery. Few health workers expressed their grievances on the transportation allowances they received during the trainings and supervision meetings. They mentioned that unlike Nepal government’s system, from the implementing NGO, they did not receive travel allowances based on the distance travelled.

The health workers perceived the positive response from the community as a good in-kind incentive which motivated them to work better. However, they still expected financial incentives to take on additional responsibility of mental health service delivery, as evident from the interview excerpt with a male prescriber: *“Motivation means refresher training of 1-2 days. We would learn new things and also get incentives. If we get incentives, we would also feel enthusiastic to work”.*

#### Stigma causing dropouts

The high community stigma towards mental health problems was identified as the key barrier responsible for default and dropouts. A FCHV said, *“Some do not visit heath post because of the fear of stigma and discrimination. They feel ashamed to go to health centre since they fear somebody might see them going to health centre for mental health treatment”.*

Stigma (including perceived stigma) was also thought to be a barrier for treatment effectiveness and recovery as people discontinue medicine with the fear of somebody seeing them taking medicine for mental health problems. A FCHV said, *“Though we tell them not to discontinue medication, they do not listen to us and do according to their own wish. I don’t know whether we are not being able to make them understand or they are being careless?”*

### Strategies to overcome the barriers

#### Ensure dedicated staff available at health facility

The health workers were of the opinion that there should be a policy level decision that at least one prescriber need to be available at the health post at all times. A male prescriber said*, “When the DPHO [District Public Health Office] organizes any training, it should be well-managed so that at least one prescriber is present at the health post. They should not be calling everyone at the same time”.*

Some of the respondents also suggested that among the trained primary health care workers, one health worker should be appointed as mental health focal person to support and monitor mental health service delivery. A male prescriber said*, “A focal person should be appointed separately for mental health. If focal person is not appointed by the central level then nobody would work properly. Appointment of the person should be in written form otherwise nobody would fulfill the responsibility”.*

#### Allocate dedicated and confidential space for counselling

Separate counselling space was suggested by the respondents as a strategy to ensure the privacy and confidentially of the patients and their family members. Respondents thought that a dedicated space for counseling would be important for quality psychosocial support as counseling involves intense conversation upon personal issues and problems, that the patients would not want to disclose in public. A non-prescriber said, *“We need medicines, and also we need counselors. We need counseling room as well because of the issue of confidentiality”.*

#### Improve on incentives and motivational benefits to existing health staff

Health workers were of the opinion that as they were doing extra work for mental health component, the government should consider providing them some financial incentives. A male prescriber said, *“if health workers are doing an additional work not considering even day and night and providing services to the people, they must get some benefits in return like some extra facilities which also include the incentive part as well”.*

For FCHVs the issue of incentives was more of an issue as they were volunteers themselves and had to do more work for mental health*.* One of the FCHVs expressed this by saying, *“We have to spend a lot of time while dealing with one client. When we go to visit some person’s house, it takes 2/3hours for counseling the client........Therefore, we also feel discouraged to work sometimes since we are not getting anything. Sometimes I feel, I simply wasted my time like” Raat bhari karayo dakshina harayo”[literal translation: shouted all night long and lost the collected alms, meaning worked hard all day long but did not get anything].*

#### Organize policy level advocacy for mental health

For the sustainability of the program the health workers were of the opinion that there should be strong policy advocacy to ensure that government takes the responsibility of implementing mental health programs in primary health care. A male prescriber elaborated this by saying: “*This program [pilot mental health project] will not be effective until it is circulated [included] as one of the prioritized national level program in the policy. Planners and policy makers play a vital role in changing the whole system so they are responsible to bring a change in the field of mental health”.*

Some respondents suggested having more involvement of patients and family members in mental health training and advocacy. A FCHV suggested, *“It would be more effective if patients and family members of patients are also provided training on mental health and make them understand about the disease”.*

#### Improve medicine supply chain management

Health workers suggested setting the minimum criteria for keeping the stock of medicines. They also suggested that the exact quantity demanded by the health facilities should be supplied to avoid the problem of stock out. An information system of drug demand and drug availability needs to be implemented as mentioned by the male prescriber who said, *“Prior information should be given. Health post has to check the stock timely. The health post should be informed about the stock of medicine”.* Some of the health workers thought that the whole system of drug supply chain needs to be improved because the unavailability of medicine was not only in the health facilities but also at the district level. A prescriber said, *“When there is no medicine, we have to go and get it. But when we go there [district public health office], it is not available there also”.*

#### Improve supervision system

Some prescribers expected more case discussion during supervision meeting but they did not get as expected. *“Supervision system is all right not perfectly well done; discussion part is found less”.* Some respondents wanted more discussion on new and difficult cases. This sentiment was represented by a prescriber who said, *“In that [current] supervision people discuss about the old cases and that’s not helpful at all. We should discuss about new cases, about cases that’s confusing so that we can correct ourselves, we can learn from it”.*

Most of the health workers suggested for monthly supervision, rather than once in 2 months. A non-prescriber said, *“What I feel is that it must be done in monthly basis like before. We have many other works also so we may forget the cases if we wait for two months for sharing”.* The on-the-spot supervision of each health facility was suggested by some health workers. For example, a female prescriber said, *“If they [supervisors] can come to each of our health post and look at our cases and provide us feedbacks then it would be even better”.*

#### Improve the mental health data collection procedures

Some health workers suggested including follow up cases *“It would have been better if we have included the total number of male–female for follow up for each disorder because there is not always the new case. So in new HMIS it must be added”.* Some others thought that the format of the HMIS form could be similar to the Tuberculosis and leprosy forms. A male prescriber said, *“Data of male and female is clearly mentioned in the present HMIS. However, it should be like the data of TB and leprosy where we can see the treatment outcome and find out whether the patient has completed the treatment or not. If we make it similar to leprosy and TB [forms] we can find out how many people got treatment services and out of them how many cases were defaulter and left the treatment. If we do like this, it would be easier for us to analyze the annual data”.*

#### Strengthen the referral system

The respondents suggested that the current referral system can be strengthened by making greater coordination with the psychiatrists at Bharatpur Hospital and instituting the process of back referral (referral from Bharatpur Hospital to Primary Health Care Facilities). A prescriber said, “They *[patients/caregivers] complain that they did not find the doctor in the place where we had referred the patient. Therefore, coordination has to be increased I think. The relationship should be strengthened so that there won’t be problem in referral”.*

## Discussion

This paper explored factors that affect the implementation of mhGAP based mental health services in primary health care setting in Nepal. The findings indicate that primary health care workers were generally supportive to the components of a newly introduced mental health care package and identified both facilitating factors as well as barriers and provided suggestions on how such barriers could be addressed. Below we discuss main findings and their implications to scaling up mental health services in primary health care setting of low resource countries like Nepal.

The provision of regular training and supervision by the specialists resulted in learning and continuous capacity building opportunities among the primary health care workers. Contrary to one-off trainings the approach of refresher trainings and regular supervision provides the opportunity to practice, ask questions to clarify confusions and get support from specialists to deal with the difficult cases. This builds up the primary health care workers’ confidence in the diagnosis and treatment of common mental health problems. The importance of regular training and supervision in maintaining quality of mental health and psychosocial services is well documented in the literature. For example, in Rwanda the training and regular mentorship of primary health care nurses along with system based improvements was found to be a potential model of integrating mental health into primary health care [10]. A model of decentralization of mental health care was implemented in Ethiopia where nurses were trained and supervised to provide drug prescription as well as initiated community based mental health activities on awareness and education [11].

Our study, however, shows that the full potential of the trained health workers could not be utilized due to frequent transfer of primary health care workers. As a result of transfer system, there was double loss as health workers who were trained in mental health got transferred to other health facilities which did not have mental health services so they could not practice the knowledge and skills they learnt during training and supervision provided by the specialists. This also created capacity crunch at the health facility from which the trained health workers were transferred and replaced by new health workers who did not receive mental health training. This is only a problem now as the integration of mental health care in primary health care has not been rolled out yet across the country. Once, it is done, the staff turnover is not so much of a problem as people transferred from one health facility to the next would be able to practice their knowledge and skills in delivering mental health services. The transfer system was a barrier for providing MH services because mental health training of the new health workers was not always possible due to financial constraints and as a consequence there was more workload for the remaining health workers. A similar finding of high staff turnover was reported in a study conducted in Africa and Asia, including Nepal [17]. Some of those trained health workers also travel out of the district for official work, training, workshops and sometimes are on long leave, leaving behind a vacuum for the delivery of mental health services. When patients come with expectation of treatment and do not find trained health workers, they lose trust with health facilities and do not return again for mental health services.

The issue of trust in services was clearly impacted by the frequent stock-out of psychotropic drugs in some health facilities. Even after several visits, the patients could not get psychotropic drugs. This resulted into frustration and therefore patients did not return to the health facility. The supply of psychotropic drugs itself and lack of storage facilities were thought to be the challenges in the stock-out of psychotropic drugs. Other studies in Nepal also report unavailability of psychotropic drugs in health facilities [12, 29, 30]. All these findings indicate that there is a need to strengthen overall psychotropic drug supply chain from the national to the health facility level. At the health facility level the delivery, storage and distribution aspect of psychotropic drug supply chain need strengthening. The system of buffer stock at the district level could be one of the options to address the stock-out of psychotropic drugs at the health facilities. The information system on psychotropic drugs demand, supply and availability needs to be further strengthened by a sound monitoring system that collects data on the several levels of the psychotropic drug supply chain and analyzes data to improve the distribution of psychotropic drugs to health facilities based on the available stock, the client flow and drug demand.

The availability of guidelines, treatment protocols and IEC materials in Nepali language facilitated health workers’ effort in providing mental health services. Often in developing countries there is lack of mental health documents published in the local language so the primary health care workers have to refer to documents in English. But, due to limited English language proficiency many primary health care workers do not manage to learn from English documents. Therefore, availability of mental health related documents in Nepali language was a facilitating factor to improve the knowledge, skills and expertise needed to provide better mental health services. Similarly, they felt comfortable educating the patients about mental health because of the pictorial posters, charts and banners available in local language. The availability of culturally appropriate materials in local language was thought to be crucial for addressing stigma and mental health treatment gap [31]. However, our study showed that only availability of the materials did not increase the use of the materials, especially when health workers were not motivated to provide mental health services due to lack of sufficient incentives.

Some health workers thought that after the introduction of mental health services in the health facility, they had to do additional work so they should be compensated through financial or non-financial benefits. The issue of workload came frequently during the interview which suggests that it might affect the helping relationship between the health workers and people with MNS disorders. Hence, to improve mental health care relationship, there is a need to embrace the quadruple aim of health care as suggested by Bodenheimer and Sinsky [32]. The quadruple aim added one more component (improving the work life of the health workers) to the already existing triple aims (enhancing patients’ experience, improving population health, reducing cost). Earlier, triple aim was thought to be the best framework to assess health system performance. But, lately, scholars argued that the fourth aim is a foundational element which helps other aims to be realized [33].

Past studies have also documented the issue of workload. For example, the health workers work burden was one of the main challenges experienced while developing a district mental health care package in Nepal [29]. Similarly, another study from Nepal showed that task-shifting in mental health risks over-burdening the health workers and therefore calls for compensation for all those involved in task-shifting [34]. Therefore, certain level of incentives might need to be put in place. Examples of such incentives could be, improving working conditions, providing financial incentives, social acknowledgment and providing opportunities for career development as suggested by a systematic review on health workers’ motivation in low and middle income countries [35]. Improving work environment for health workers and providing an opportunity for them to find joy and meaning in work will, therefore, contribute in achieving the triple aim of health system performance [33], which will ultimately contribute to integration of mental health into primary health care. Provision of connecting primary health care workers with community structures through community linkages, referral pathways, defaulters tracking system and home based care helped bridge the gap between demand and supply side of mental health service delivery. The engagement of the community and their participation in the detection of potential mental health problems and referral to the nearby health facility was found to be feasible and effective in a study conducted in Chitwan district of Nepal [25–27]. When mental health is integrated in primary care, linkage with community based services is necessary because it aids early identification, referral to appropriate service providers for treatment and community level post-treatment follow up. The study findings also suggest that the macro (system level), meso (organization level) and micro (clinical level) model of integration suggested by Valentijn and colleagues [13] might be helpful in addressing barriers and strengthening the facilitating factors for mental health integration in primary health care setting.

Regular community engagement and educational programs also help in reducing stigma related to mental illness [36]. Despite reduction of stigma due to community mental health awareness program, mental illness is still stigmatized in Nepali communities. In Nepali society mental illness is directly attributed to the “broken mind” and its treatment is thought to be only for the so called “mad people” [37].. The social stigma was the main factor preventing people from visiting the health facilities for mental health treatment and follow up. Health workers attributed the increased dropout of mental health service users to stigma associated with patients of mental health problems and their families. A qualitative study from Nepal also showed that stigma and negative cultural norms were responsible for reduced access and demand for mental health services [38]. Along with other anti-stigma programs, the involvement of recovered patients and their family members in mental health awareness raising and advocacy could help bring positive mental health reforms as it did in Zambia [8].

A functioning information system to document patients’ demographic and treatment records, stock of medicine and incoming and outgoing referrals was found to be a facilitating factor for integration of mental health in primary health care. Patients’ record keeping has enabled the health workers to understand the pattern of symptoms among the patients and eventually track the progress of the treatment process, follow up procedures as well as stock of medicines. However, just setting up information system does not guarantee its success. For example, currently the information is collected at health facility and shared at the district level to report to the health management information system. But, the analysis of the data at the health facility level is rarely done to reflect on trend of patient flow, referrals, treatment adherence and drop outs/defaulters and develop appropriate corrective actions. The flow of data goes only upwards to report the performances as opposed to be used at the health facility level using data driven continuous quality improvement. The quality and usefulness of the system largely depends up on its proper implementation and maintenance [39]. Therefore, the focus needs to be on the analysis and use of the data at the point of collection [40].

Integration of psychosocial support and counseling in the primary health care facilities contribute to the effects of the treatment of common mental disorders and prevention of day to day distress of patients and family members. However, the lack of private space for counseling in some health facilities compelled health workers to provide counseling services in several open spaces where the privacy and confidentially could not be ensured. The counseling intervention requires a healing environment that comprise of activities, systems and physical setting [41]. Only a private, safe and pleasant space can provide such healing environment which is a prerequisite for effective recovery from mental illness. A previous study in Nepal has also documented positive results of combining psychotropic medication and counselling services for a complete recovery of people with mental health problems [42].

### Strengths and limitations

The strength of the study is that it includes the perspectives of all three categories of primary health care workers involved in mental health service delivery, so the findings are representative for the development and strengthening of the multi layered provision of community based mental health and psychosocial interventions in districts where tertiary mental health services are available. However, the findings cannot be generalized for other rural districts of Nepal because Chitwan district is much advanced in terms of the availability of mental health care where tertiary care is available from government and private hospitals and primary mental health care is being made available through PRIME project. One of the limitations of the study is that the researchers were from the same organization that provided the mental health training and other mental health system strengthening support, so this might have influenced the primary health care workers to provide socially desirable answers. The research team, however, tried its best to minimize the respondent bias by explaining them that it is not the evaluation of health workers’ performance rather the research team was interested to hear the perspectives of health workers on what went well and what did not and what needs to be done in future to improve on the issues/ components that did not go well.

## Conclusions

Factors supporting integrated mental health service delivery included availability of protocol and guidelines, provision of regular training, supervision and coaching system, established referral system, system for patients information management, the component of community engagement, provision of home based care and follow up, the provision of psychosocial support along with drugs and various level of coordination with government, non-governmental and community structures. In terms of barriers, health workforce related barriers included the frequent transfer of trained health workers and arrival of new health workers without mental health training. Patient level challenges included the drop outs, defaulters and not going to the referred places. There were also challenges in terms of private space for counselling, stock of medicine and use of mental health information to improve the quality of mental health services.

To address the barriers for integration of mental health services in primary health care, the strategies suggested by the respondents included; policy advocacy, provision of dedicated space within health facility for counseling services, provision of buffer stock for psychotropic drugs, regular refresher training, clinical supervision and financial benefits to the health workers, strengthened referral pathways, defaulters tracking system, home visits and supervision and feedback system.

## Data Availability

The data is reported within this manuscript.

## Abbreviations

CIDT: Community Informant Detection Tool
DALY: Disability Adjusted Life Year
DPHO: District Public Health Office
EMERALD: Emerging Mental Health Systems in Low and Middle Income Countries
FCHVs: Female Community Health Volunteers
HMIS: Health Management Information System
IEC: Information, Education and Communication
LMICs: Low and Middle Income Countries
MhGAP: Mental Health Gap Action Program
MNS: Mental, Neurological and Substance Abuse
NGO: Non-Governmental Organization
OPD: Out Patient Department
PRIME: Program for Improving Mental Health Care
WHO: World Health Organization
